# Outcomes of sustained low efficiency dialysis versus continuous renal replacement therapy in critically ill adults with acute kidney injury: a cohort study

**DOI:** 10.1186/s12882-015-0123-4

**Published:** 2015-08-04

**Authors:** Abhijat Kitchlu, Neill Adhikari, Karen E. A. Burns, Jan O. Friedrich, Amit X. Garg, David Klein, Robert M. Richardson, Ron Wald

**Affiliations:** Department of Medicine, Division of Nephrology, University of Toronto, Toronto, ON Canada; Interdepartmental Division of Critical Care, University of Toronto, Toronto, ON Canada; Department of Critical Care Medicine and Sunnybrook Research Institute, Sunnybrook Health Sciences Centre, Toronto, ON Canada; Li Ka Shing Knowledge Institute of St. Michael’s Hospital, 61 Queen Street East, 9-140, Toronto, ON M5C 2 T2 Canada; Departments of Critical Care and Medicine, St. Michael’s Hospital, Toronto, ON Canada; Division of Nephrology, Department of Medicine, University of Western Ontario, London, ON Canada; Department of Epidemiology & Biostatistics, University of Western Ontario, London, ON Canada

## Abstract

**Background:**

Sustained low efficiency dialysis (SLED) is increasingly used as a renal replacement modality in critically ill patients with acute kidney injury (AKI) and hemodynamic instability. SLED may reduce the hemodynamic perturbations of intermittent hemodialysis, while obviating the resource demands of CRRT. Although SLED is being increasingly used, few studies have evaluated its impact on clinical outcomes.

**Methods:**

We conducted a cohort study comparing SLED (target 8 h/session, blood flow 200 mL/min, predominantly without anticoagulation) to CRRT in four ICUs at an academic medical centre. The primary outcome was mortality 30 days after RRT initiation, adjusted for demographics, comorbidity, baseline kidney function, and Sequential Organ Failure Assessment score. Secondary outcomes were persistent RRT dependence at 30 days and early clinical deterioration, defined as a rise in SOFA score or death 48 h after starting RRT.

**Results:**

We identified 158 patients who initiated treatment with CRRT and 74 with SLED. Mortality at 30 days was 54 % and 61 % among SLED- and CRRT-treated patients, respectively [adjusted odds ratio (OR) 1.07, 95 % CI 0.56–2.03, as compared with CRRT]. Among SLED recipients, the risk of RRT dependence at 30 days (adjusted OR 1.36, 95 % CI 0.51–3.57) and early clinical deterioration (adjusted OR 0.73, 95 % CI 0.40–1.34) were not different as compared to patients who initiated CRRT.

**Conclusions:**

Notwithstanding the limitations of this small non-randomized study, we found similar clinical outcomes for patients treated with SLED and CRRT. While we await the completion of a trial that will definitively assess the non-inferiority of SLED as compared to CRRT, SLED appears to be an acceptable alternative form of renal support in hemodynamically unstable patients with AKI.

**Electronic supplementary material:**

The online version of this article (doi:10.1186/s12882-015-0123-4) contains supplementary material, which is available to authorized users.

## Background

There is ongoing controversy about the optimal modality for the delivery of renal replacement therapy (RRT) to critically ill patients with acute kidney injury (AKI). Continuous renal replacement therapy (CRRT), which permits gradual fluid and solute removal, has been associated with greater hemodynamic stability and a higher likelihood of kidney recovery as compared to standard intermittent hemodialysis [1–3]. However, the logistic burdens of administering CRRT, including the need for anticoagulation and specialized pre-manufactured solutions, and overall higher costs, as well as the need to frequently interrupt therapy to allow for off-unit testing and procedures, detract from the theoretical benefits of this modality [4, 5]. Moreover, randomized trials have not demonstrated better patient- or kidney- survival with CRRT as compared to conventional intermittent dialysis [6].

Sustained low-efficiency dialysis (SLED), in which conventional hemodialysis machines are used to provide extended duration RRT (8 – 12 h vs 3-4 h with classic intermittent hemodialysis), has emerged as an alternative to CRRT for patients with hemodynamic instability. Whereas CRRT often necessitates some form of anticoagulation to prevent filter clotting, SLED may be readily performed with no anticoagulation. A session of SLED, especially if performed during the overnight hours, may be scheduled around tests and procedures and is thus less likely to be interrupted. Observational studies have suggested comparable clinical outcomes, and in some cases lower costs, in patients treated with SLED as compared to CRRT [7–12]. A recently published randomized trial of SLED vs CRRT that focused on clinical outcomes was difficult to interpret as the two forms of RRT were administered in an unconventional manner [13].

We conducted a retrospective cohort study comparing SLED and CRRT using a comprehensive registry of critically ill adults with AKI who received RRT. The primary outcome was all-cause mortality at 30 days after RRT initiation.

## Methods

### Data sources and study population

Patients were identified from the St. Michael’s Hospital Acute Kidney Injury Registry, which comprises all adults who commenced RRT for AKI in the hospital’s four intensive care units (medical-surgical, cardiovascular, trauma-neurosurgical or coronary care), between April 2007 and September 2012. AKI was defined by the Acute Kidney Injury Network (AKIN) criteria [14]: minimum creatinine increase of 27 μmol/L or 50 % rise from baseline (if baseline ≥ 354, then a rise of 44 μmol/L qualified for a diagnosis of AKI). Patients with pre-existing end-stage renal disease who were erroneously entered in the Registry were excluded. We also excluded patients who initiated RRT using intermittent hemodialysis (IHD) because at our institution, IHD is reserved for patients who are hemodynamically stable whereas SLED or CRRT are deployed in patients with perceived hemodynamic instability. We restricted this analysis to patients for whom we could ascertain vital status at 30 days following the initiation of RRT.

Our study was approved by the Research Ethics Board of St. Michael’s Hospital. The need for patient-level consent was waived by the Research Ethics Board.

### Exposure definition

Initial RRT modality, SLED versus CRRT, was the exposure of interest. Choice of initial RRT modality was made at the discretion of the consulting nephrologist in collaboration with the attending critical care physician. In the absence of compelling clinical or personnel concerns, hospital policy discouraged switches between CRRT and SLED.

### Description of administered therapies

CRRT was administered by ICU nurses as continuous venovenous hemodiafiltration or continuous venovenous hemofiltration using Prisma and Prismaflex (Gambro, Richmond Hill, ON) devices. AN-69-based filters were utilized, with blood flows ranging from 100 – 200 mL/hr and target effluent rates of 20 – 35 mL/kg/hr.

SLED was introduced as an alternative to CRRT in 2007. Both modalities were targeted to hemodynamically unstable patients. SLED was delivered by dialysis nurses using Phoenix^TM^ dialysis machines (Gambro, Richmond Hill, ON) and CA210 or Xenium 210 dialyzers (Baxter, Deerfield, IL). SLED sessions were targeted to 8 h in duration (minimum 6 h) with blood and dialysate flows of 200 and 350 mL/min, respectively. The minimum frequency of SLED treatments was three times per week but could be increased at the nephrology team’s discretion.

The nephrology consultation service, comprising trainees who were supervised by an attending nephrologist, prescribed all CRRT and SLED treatments.

### Data collection and baseline characteristics

We obtained the following data from the St. Michael’s Hospital Acute Kidney Injury Registry: patient age and gender; reason for ICU admission; pre-morbid kidney function (derived from the abbreviated Modification of Diet in Renal Disease formula [15] using the last available pre-hospitalization serum creatinine); Charlson comorbidity score [16]; and the following parameters at the time of RRT initiation: basic biochemistry and hematologic values; urine output, receipt of mechanical ventilation, requirement for vasopressors and the Sequential Organ Failure Assessment (SOFA) score [17] (as modified for use in the Registry [18], see Additional file 1) at the time of RRT initiation. The SOFA score was also recorded at 48 h following RRT initiation.

### Outcomes

The primary outcome was all-cause mortality at 30 days following RRT initiation. Secondary outcomes included RRT dependence at 30 days, cumulative fluid removal seven days following initiation of RRT, and early clinical deterioration, defined as death or increase in SOFA score within 48 h of RRT initiation)

### Statistical analyses

We expressed continuous variables as means [standard deviations (SD)] or medians [interquartile range (IQR)], as appropriate, and categorical variables as numbers (percentages). The characteristics of patients who initiated SLED vs CRRT were compared using the *t*-test, Mann-Whitney *U* test or Fisher’s exact test. Logistic regression was used to evaluate the relationship between RRT modality (SLED versus CRRT) and the outcomes of interest. For the primary outcome of mortality at 30 days, we adjusted for age, sex, ICU type, Charlson score, mechanical ventilation and vasopressor status, serum creatinine at the time of RRT initiation, SOFA score and urine output at the time of RRT initiation. These covariates were chosen based on clinical relevance. For the secondary outcomes for which there were fewer events, we adjusted our models for baseline serum creatinine, SOFA score, urine output at the time of RRT initiation and Charlson score.

### Additional analyses

We examined the primary outcome in a subgroup analysis wherein we stratified patients by initial SOFA score (less than vs greater than or equal to the median value of 16) to determine whether severity of illness modified the relationship between modality and 30-day mortality. Since switches in RRT modality (eg, SLED to CRRT) may misclassify the exposure and dilute the association between initial RRT modality and clinical outcomes, we conducted two sensitivity analyses. In the first, we designated RRT modality as the one used for the majority of treatment sessions received from the initiation of RRT through Day 30. In the second, we restricted the cohort to patients who remained on a consistent modality for the first three RRT sessions.

We performed all analyses using SAS software version 9.1.3 (SAS Institute Inc., Cary, NC).

## Results

**Fig. 1:**
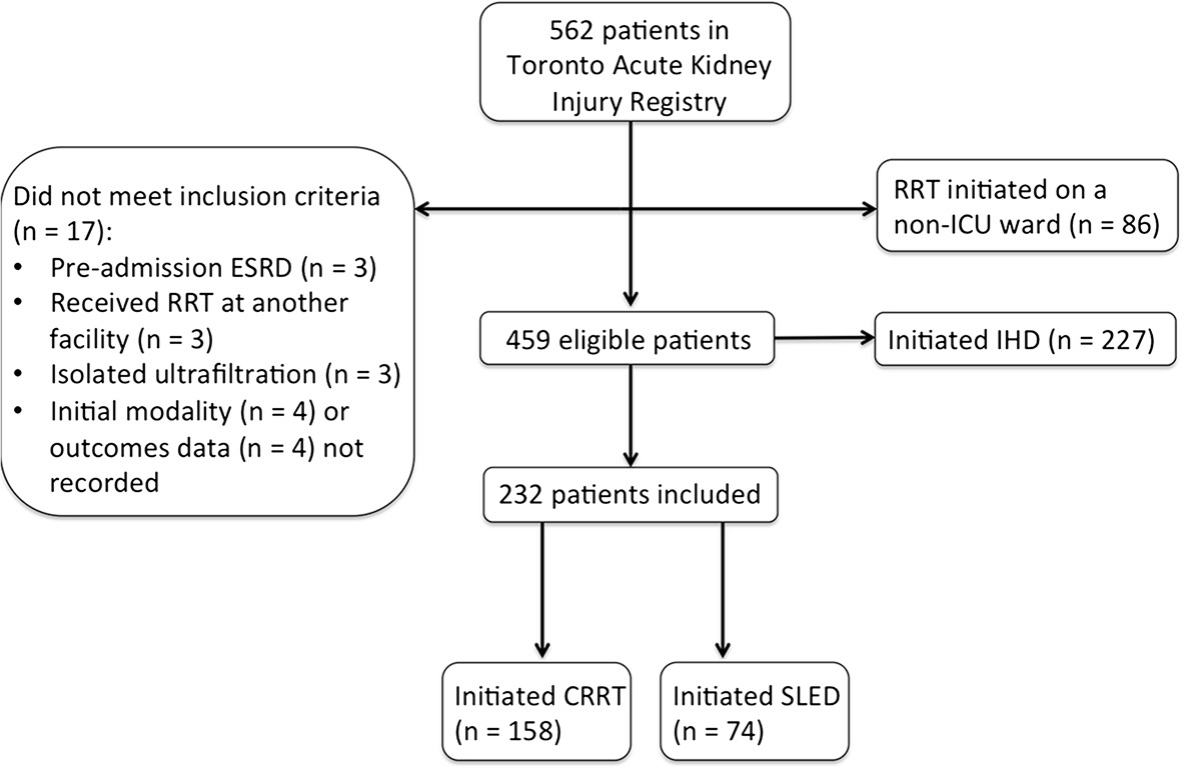
Study Flow Diagram

The steps that led to assembly of the analytic cohort are presented in Fig. 1. We identified 562 patients within our AKI Registry at the time of analysis. Of these, we excluded 227 patients who initiated RRT with IHD and 86 patients received their first RRT treatment in a non-ICU setting. Other reasons for exclusion were the mistaken inclusion of patients commencing RRT for ESRD (n = 3); RRT initiation at an outside facility (n = 3); the receipt of isolated ultrafiltration as the initial RRT modality (n = 3); or missing information on initial modality or outcome (n = 8) (Fig. 1). Among the remaining 232 patients, 158 patients received CRRT as their initial RRT modality, and 74 received SLED.

### Baseline characteristics

Baseline characteristics, stratified by initial receipt of SLED or CRRT, are presented in Table 1. Patients who initiated CRRT had higher Charlson (2.61 ± 2.53 versus 1.79 ± 1.77 for SLED recipients, *p* = 0.03) and SOFA(16.4 ± 3.08 versus 15.4 ± 3.65 for SLED recipients, *p* = 0.03) scores at the time of RRT initiation and were more likely to be mechanically ventilated (96.5 versus 85.6 % of SLED recipients, *p =* 0.03).

**Table 1:** Baseline characteristics

Variable	CRRT (n = 158)	SLED (n = 74)	*p* Value
Age at initiation of RRT	62.1 ± 15.3	60.6 ± 17.3	0.50
Male (%)	94 (59.5)	50 (67.6)	0.25
Transferred from another institution (%)	65 (41.1)	35 (47.3)	0.40
Surgical admission (%)	91 (57.6)	34 (46.0)	0.12
ICU Type			0.93
Medical-Surgical/Neurosurgical/Trauma	68.5	68.9	
Cardiovascular/CCU	31.5	31.1	
Cardiac surgery	42 (26.6)	13 (17.6)	0.14
AAA repair	5 (3.16)	3 (4.05)	0.71
Mean Charlson score	2.61 ± 2.54	1.80 ± 1.77	0.03
Charlson score (categories)			
0	28 (17.7)	18 (24.3)	0.22
1	34 (21.5)	20 (27.0)	
≥2	96 (60.8)	36 (48.7)	
Mechanical ventilation	151 (95.6)	64 (86.5)	0.03
Vasopressors	138 (87.3)	59 (79.7)	0.17
SOFA score	16.4 ± 3.08	15.4 ± 3.65	0.03
Pre-morbid serum creatinine^a^, μmol/L	130.7 ± 91.7	135.3 ± 83.8	0.57
Admission creatinine, μmol/L	185.7 ± 181.5	214.0 ± 202.1	0.75
Admission BUN, mmol/L	13.5 ± 10.8	14.0 ± 11.7	0.89
ICU admission creatinine, μmol/L	225.6 ± 173.4	259.6 ± 192.1	0.11
Creatinine, μmol/L	328.9 ± 136.1	365.1 ± 146.7	0.07
BUN, mmol/L	23.1 ± 11.7	22.3 ± 12.5	0.68
Proportion with BUN ≥ 40 mmol/L	15 (10.3)	7 (11.1)	0.87
SBP, mmHg	109.3 ± 20.8	110.9 ± 17.5	0.56
DBP, mmHg	54.5 ± 11.3	57.2 ± 11.5	0.10
Urine output on day of RRT initiation, mL	328.8 ± 580.5	387.6 ± 607.0	0.48
Urine output < 400 mL/24 hrs	126 (80.0)	51 (68.9)	0.07
Hemoglobin, g/L	85.9 ± 14.2	89.7 ± 20.0	0.15
WBC, x 10^9^/L	16.4 ± 11.4	16.7 ± 18.3	0.89
Platelets, x 10^9^/L	134.0 ± 103.7	153.6 ± 104.2	0.18
Sodium, mmol/L	137.7 ± 5.92	136.8 ± 6.80	0.31
Potassium, mmol/L	4.60 ± 0.84	4.65 ± 0.80	0.69
Proportion with potassium ≥ 6 mmol/L	12 (7.6)	5 (6.8)	0.82
Bicarbonate, mmol/L	18.6 ± 5.5	19.2 ± 5.0	0.43
Proportion with bicarbonate < 15 mmol/L	32 (20.2)	12 (16.2)	0.46
Glucose, mmol/L	7.73 ± 3.38	7.71 ± 3.35	0.96
Lactate, mmol/L	4.62 ± 4.76	4.50 ± 5.13	0.87
Bilirubin, mmol/L	59.9 ± 80.2	81.9 ± 122.9	0.19
pH, arterial	7.29 ± 0.16	7.32 ± 0.15	0.19

Continuous data are presented as means ± standard deviation and categorical data as number (%). Clinical or laboratory parameters were measured at initiation of RRT unless otherwise specified. ^a^Patients for whom data not available were excluded from the computed means

### RRT Treatment characteristics

We examined 1107 treatment sessions (698 CRRT and 509 SLED sessions) to characterize the RRT delivered to members of the cohort. The median duration of SLED sessions was 7.11 h (IQR 6.00–7.92). On average, 91.9 % (SD 1.7 %) of the prescribed SLED treatment duration was delivered. The mean proportion of prescribed CRRT time that was actually delivered was 84.8 % (SD 22.0 %), which is compatible with 20.3 h in a 24-h period. SLED was delivered without use of anticoagulation in 86 % of treatment sessions, compared to 6 % of CRRT sessions. Further information on the delivered therapies is shown in Table 2.

**Table 2:** Descriptors of RRT treatments

Variable	CRRT	SLED
Number of treatments	698	409
Median treatment time (hrs) [IQR]	20.3 [19.0—24.0]	7.11 [6.00—7.92]
Mean proportion of prescribed treatment time delivered (hrs) [SD]	0.85 [0.22]	0.92 [0.17]
Mean blood flow rate (mL/min)	160.2 [56.2]	216.9 [30.1]
Mean effluent flow (mL/kg/hr)	27.2 [10.7]	---
Anticoagulation^a^ (%):		
heparin	166 (24.7)	56 (13.8)
citrate	469 (69.7)	0 (0.00)
none	38 (5.6)	350 (86.2)

Per treatment analysis (each patient could have received multiple treatment sessions)

^a^Data not available for 28 treatments (25 CRRT and 3 SLED)

### Outcomes

**Fig. 2:**
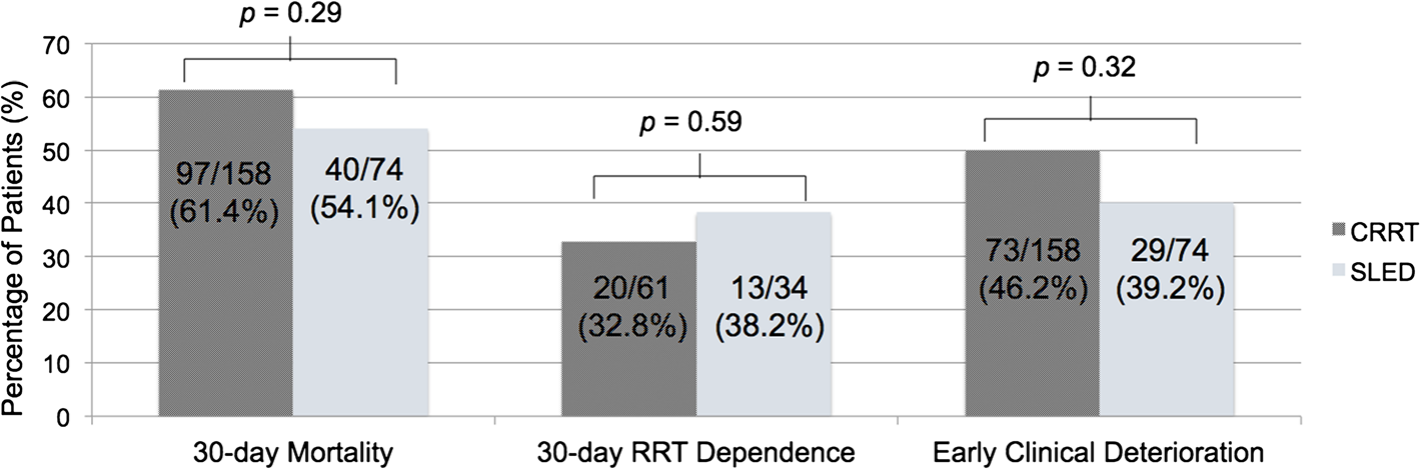
30-day Mortality, RRT Dependence and Early Clinical Deterioration by RRT Modality

All-cause mortality at 30 days was 54 % and 61 % among SLED- and CRRT-treated patients, respectively. After adjustment for possible confounders, SLED was not associated with 30-day mortality (adjusted OR 1.07, 95 % CI 0.56–2.03), as compared with CRRT (Table 3, Fig. 2). Additionally, there were no differences in secondary outcomes between the groups, including the adjusted risk of RRT dependence at 30 days and clinical deterioration within 48 h of RRT initiation (Table 4). Cumulative fluid removal at seven days was not significantly different between the groups (5846.5 ± 8855.6 mL versus 8180.2 ± 11322.0 mL, respectively).

**Table 3:** Mortality at 30 days

Variable	Unadjusted odds ratio (95 % CI)	Adjusted odds ratio (95 % CI)
SLED (versus CRRT)	0.74 (0.42–1.29)	1.07 (0.56–2.03)
Age	1.00 (0.98–1.01)	1.01 (0.99–1.03)
Male	0.74 (0.43–1.27)	0.85 (0.45–1.59)
Medical-Surgical or Trauma-Neurosurgical ICU vs Cardiovascular/CCU	1.29 (0.74–2.26)	1.10 (0.57–2.12)
Charlson score	1.08 (0.96–1.21)	1.12 (0.97–1.29)
SOFA score at RRT initiation	1.27 (1.16–1.39)	1.37 (1.20–1.56)
Mechanical ventilation	1.31 (0.49–3.52)	0.67 (0.20–2.27)
Receiving vasopressor	1.65 (0.80–3.40)	0.44 (0.17–1.14)
Serum creatinine at RRT initiation, per 50 μmol/L	0.87 (0.79-0.96)	0.93 (0.83-1.05)
Urine output at RRT initiation, per 100 mL/day	0.97 (0.93–1.02)	0.98 (0.93-1.03)

**Table 4:** Secondary outcomes, subgroup and sensitivity analyses

Secondary outcome	Unadjusted odds ratio (95 % CI)	Adjusted odds ratio (95 % CI)
RRT dependence at 30 days	1.27 (0.53–3.04)	1.36 (0.51–3.57)
Composite outcome: increase in SOFA score or death at 48 h	0.77 (0.44–1.35)	0.73 (0.40–1.34)
Subgroup analysis^a^		Adjusted odds ratio (95 % CI)
SOFA score ≥16		1.44 (0.52–3.95)
SOFA score <16		0.84 (0.34–2.11)
Sensitivity analysis^b^		
SLED as predominant RRT modality		1.70 (0.75–3.82)
SLED as exclusive RRT modality in first 3 treatment sessions		1.36 (0.61–3.82)

^a^30-day mortality for patients stratified by SOFA score

^b^30-day mortality for patients with exposure defined by modality of majority of RRT treatment sessions received and patients with no change in RRT modality within the initial three RRT sessions

### Subgroup and sensitivity analyses

There was no association between RRT modality and mortality within strata of acute illness severity (SOFA ≥ vs. < 16, Table 4).

We repeated the primary analysis with re-categorization of patients according to the RRT modality received for the majority of treatment sessions during the first 30 days. SLED was not significantly associated with 30-day mortality [adjusted OR 1.70, 95 % CI 0.76–3.28]. Similarly, after restricting our cohort to patients who received a consistent modality during the first three treatment sessions (n = 183), we did not identify a relationship between SLED and 30-day mortality [adjusted OR 1.36, 95 % CI 0.61–3.82] (Table 4).

## Discussion

In critically ill patients with AKI and hemodynamic instability, the initiation of RRT with SLED is not associated with a different short-term risk of death and RRT dependence as compared to the use of CRRT. These findings were robust after adjustment for demographics, comorbidities, illness severity, and baseline renal function and in various sensitivity analyses.

Our results are congruent with those of previous observational studies. Marshall *et al* used a time-series approach to examine mortality rates after a unit-wide switch at 3 hospitals from CRRT to SLED (referred to as ‘Prolonged Intermittent Renal Replacement Therapy’ or PIRRT in that study) with SLED administered using comparable parameters to those described in this study. The transition to SLED was not associated with increased mortality [8]. Sun *et al* completed a retrospective analysis of 65 patients treated with CRRT and 80 treated with SLED and also observed no significant difference in 60-day mortality. There was, however, a higher likelihood of RRT independence in the CRRT group [19].

Two randomized trials have compared SLED and CRRT though their findings do not clearly demonstrate the superiority of either modality. Abe *et al* randomized 60 patients to SLED or CRRT and found no significant difference in survival to ICU discharge or 30-day survival (whichever occurred first) [20]. In-hospital renal recovery was not affected by RRT modality. In the largest clinical trial to compare SLED and CRRT with respect to clinical outcomes, Schwenger *et al* randomized 232 critically ill patients with AKI to CRRT or SLED and found no difference in survival [13]. Importantly, the mean SLED and CRRT durations achieved were similar at 14.9 ± 4.4 hrs/session and 15.9 ± 4.2 hrs/session, respectively, which are atypical for the modalities being compared. In the current study, treatment durations for SLED and CRRT differed substantially, (7.10 ± 1.9 hrs and 20.3 ± 5.3 hrs, respectively) and align well with traditional descriptions of SLED and CRRT [7, 9, 12]. In our CRRT group, the mean effluent prescribed was 27 mL/kg/hr which is compatible with prevailing recommendations [21].

### Kidney recovery, fluid removal and anticoagulation

It has been hypothesized that after AKI, autoregulation of blood flow is impaired, with susceptibility to further kidney damage as a result of dialysis-related hemodynamic instability [22, 23]. CRRT has the purported benefit of improved hemodynamic tolerability compared to IHD [1, 2, 24]. Long-term kidney outcomes may also be better among patients who received CRRT [24–26]. Although we demonstrated that surviving SLED recipients are not more likely to remain RRT-dependent as compared to CRRT recipients, our sample size is small and the relative impact of SLED on long-term kidney function is an important question for future study.

Our findings also demonstrated comparable volume control among SLED and CRRT recipients in the week after RRT initiation and support the findings of previous smaller observational studies [7, 27].

Since the need for anticoagulation often presents a series of practical challenges in critically ill patients who require RRT, our finding that the vast majority of SLED sessions could be delivered with no anticoagulation is compelling. Since clinical outcomes of SLED seem comparable to CRRT, the ability to deliver RRT without the bleeding and metabolic complications of current anticoagulation options commonly used in CRRT (e.g., heparin or regional citrate anticoagulation) may represent a major benefit of SLED. Delivery of SLED predominantly without anticoagulation has not been reported in other studies assessing this modality [13, 19, 20, 28].

### Strengths and limitations

Our study’s strengths include use of a robust database that comprises clinically relevant measures of comorbidity, baseline renal function, biochemical and hemodynamic indices, and acute illness severity. This enabled us to adjust for critical confounders of the relationship between RRT modality and outcomes and at least partially overcome bias related to factors that may influence a clinician to initiate RRT with either SLED or CRRT. Our study included assessment of early clinical deterioration (within the first 48 h after RRT initiation), which, to date has not been examined in the literature and showed that SLED recipients did not experience a more rapid clinical decline during the critical period after RRT initiation. Our study was also conducted in a centre where both SLED and CRRT were readily available treatment options. This allowed us to compare contemporaneous patients, rather than historical controls, as occurs in situations when hospitals make wholesale switches from one modality to the other. Finally, given that our study is, to our knowledge, the largest non-randomized comparison of SLED and CRRT and comprised critically ill patients with AKI from a variety of causes, we expect that our results will have broad generalizability and complement information gleaned from previous smaller studies. As such, we believe that this observational study provides vital “real world” information that encompasses a broader spectrum of patients that might be enrolled in clinical trials.

Our study also has evident limitations. As a non-randomized study, our findings are susceptible to unmeasured confounding. Given the greater pre-existing experience and comfort level with CRRT, it is possible that patients with more severe illness were treated with CRRT preferentially over SLED. This is suggested by the slightly higher SOFA and Charlson scores and the higher frequency of mechanical ventilation in the CRRT group. These statistically significant differences may bias clinical outcomes in favour of SLED. In order to mitigate the impact of confounding, we included multiple measures of pre-morbid health and acute illness severity in our multivariable model. After adjustment for these imbalances, the receipt of SLED was not associated with inferior outcomes. The primary analysis examined patients based on the initial RRT modality received; however, we recognized that patients could have been treated with both modalities, which would bias our results towards the null hypothesis (or no effect). It was therefore reassuring that our sensitivity analyses were consistent with our primary analysis. Our study was restricted to 30-day follow-up and as a result, we were unable to comment on mortality or RRT dependence associated with either modality over the long-term. It has been argued that the nephroprotective benefits of CRRT only become evident 90 days or more after initiation of RRT, once the acute illness has abated [25, 26]; however such long-term data on SLED-treated patients remains limited. Finally, although the current study is among the largest to compare SLED and CRRT with respect to clinical outcomes, our sample size is still too small to definitively evaluate whether SLED is non-inferior to CRRT.

## Conclusions

Our observational data suggest that treatment with SLED is associated with mortality and short-term renal recovery that is comparable to CRRT. Pending the completion of an adequately-powered randomized trial that will evaluate the impact of SLED on clinical outcomes, SLED appears to be an acceptable alternative to CRRT for hemodynamically unstable patients with AKI.

### Key messages

- Treatment with SLED was associated with comparable 30-day mortality and short-term RRT dependence to CRRT.
- Indicators of clinical status, including change in SOFA score within 48-h and fluid removal achieved within seven days, were similar for SLED- and CRRT-treated patients
- SLED can be readily performed for most patients without systemic or regional anticoagulation.

## Additional file

Additional file 1:
**Collection of the Modified Sequential Organ Failure Assessment (SOFA) Score.** (DOC 38 kb)

## Abbreviations

AKI: Acute kidney injury
CRRT: Continuous renal replacement therapy
RRT: Renal replacement therapy
SLED: Sustained low-efficiency dialysis
SOFA: Sequential organ failure assessment
