# Supplementary material for: Outcomes of sustained low efficiency dialysis versus continuous renal replacement therapy in critically ill adults with acute kidney injury: a cohort study
Source: BMC Nephrol. 2015 Aug 4;16:127. doi: 10.1186/s12882-015-0123-4 (PMC4522955; doi:10.1186/s12882-015-0123-4)
Supplement: Additional file 1: — Collection of the Modified Sequential Organ Failure Assessment (SOFA) Score. (DOC 38 kb) [file 12882_2015_123_MOESM1_ESM.doc]

**Appendix A – Collection of the Modified Sequential Organ Failure Assessment (SOFA) Score**

(Based on most extreme result for each category during the period of assessment.)

| | SOFA Score | 0 | 1 | 2 | 3 | 4 | | --- | --- | --- | --- | --- | --- | | ***Respiration***  **PaO_2_/FiO_2_** | ≥ 400 | ≤ 400 ≤ 300  (± resp. support) | | ≤ 200 ≤ 100  (+ resp. support) | | | ***Coagulation***  **Platelets(x 10^9^/L)** | ≥150 | ≤ 150 | ≤ 100 | ≤ 50 | ≤ 20 | | ***Liver***  **Bilirubin (µmol/L)** | < 20 | 20-32 | 33-101 | 102-204 | > 204 | | **Cardiovascular** | MAP ≥  70 mmHg | MAP < 70 mmHg | DA ≤ 5 µg/kg/min or dobutamine  or milrinone (any dose) | DA > 5 µg/kg/min or  EPI ≤ 0.1 µg/kg/min or NE ≤0.1  µg/kg/min  or VP ≤ 0.02 U/min | DA > 15 µg/kg/min or EPI > 0.1 µg/kg/min or NE > 0.1 µg/kg/min or VP ≥  0.03 U/min | | ***CNS***  **Glasgow Coma Scale (see below)** | 15 | 13-14 | 10-12 | 6-9 | < 6 | | ***Renal****  **Creatinine (µmol /L)** | < 110 | 110-170 | 171-299 | 300-440 or urine output 200-499 mL/d | ≥ 440 or urine output < 200 mL/d | |
| --- | --- | --- | --- | --- | --- | --- | --- | --- | --- | --- | --- | --- | --- | --- | --- | --- | --- | --- | --- | --- | --- | --- | --- | --- | --- | --- | --- | --- | --- | --- | --- | --- | --- | --- | --- | --- | --- | --- | --- | --- | --- | --- |

**DA= dopamine EPI= epinephrine NE= norepinephrine VP = vasopressin**

*** If patient received any form of renal replacement therapy during the day of assessment, an automatic Renal score of 4 is assigned.**

**SOFA SCORE (total 0-24):** ______________
